# Phytochemical-Based Study of Ethanolic Extract of *Saraca asoca* in Letrozole-Induced Polycystic Ovarian Syndrome in Female Adult Rats

**DOI:** 10.1021/acsomega.3c05274

**Published:** 2023-11-01

**Authors:** Na Bu, Alina Jamil, Liaqat Hussain, Abdulrahman Alshammari, Thamer H. Albekairi, Metab Alharbi, Ayesha Jamshed, Rizwan Rashid Bazmi, Anam Younas

**Affiliations:** † Department of Pharmacy, Women’s Hospital, School of Medicine, Zhejiang University, Hangzhou 31006, P. R. China; ‡ Department of Pharmacology, Faculty of Pharmaceutical Sciences, Government College University, Faisalabad 38040, Pakistan; § Department of Pharmacology and Toxicology, College of Pharmacy, King Saud University, P.O. Box 2455, Riyadh 11451, Saudi Arabia; ∥ Department of Pharmacology, Faculty of Pharmacy, Islamia University Bahawalpur, Bahawalpur 63100, Pakistan; ⊥ Department of Pharmaceutical Chemistry, Faculty of Pharmaceutical Sciences, Government College University, Faisalabad 38040, Pakistan

## Abstract

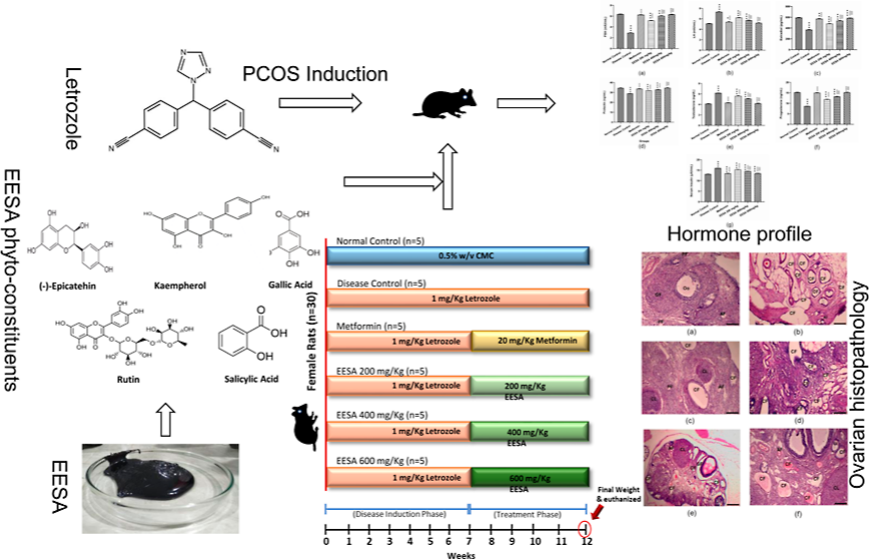

Polycystic ovarian syndrome (PCOS) is a complex metabolic and endocrine disorder which affects women of reproductive age. It is a condition in which ovaries produce an excessive amount of androgen (the male sex hormone). *Saraca asoca* (Roxb.) Willd. is a plant of the Fabaceae family. This plant has been traditionally used as a uterine tonic in leucorrhea and dysmenorrhea due to its various pharmacological activities. In this study, the ethanolic extract of *S. asoca* (EESA) was evaluated for its potential to be used for the management of PCOS. HPLC analysis revealed the presence of various phytoconstituents: kaempferol, rutin, (−)-epicatechin, salicylic acid, and gallic acid. For PCOS induction, 30 adult female rats were randomly divided into two groups: the control group (*n* = 5) and the PCOS group (*n* = 25). Letrozole (1 mg/kg/day) was administered per orally (p.o.) for a period of 7 weeks for the induction of disease. Weekly body weight measurements and daily vaginal cytology examinations were performed for disease confirmation. After disease induction, the PCOS group was further divided into five groups (*n* = 5), that is, disease control, metformin, and EESA (200, 400, and 600 mg/kg) groups, respectively, and given treatment doses for next 5 weeks. After the treatment period, all animals were weighed and euthanized humanly. Blood samples were collected for hormonal assays, lipid profiles, and liver function tests. For histological assessment of ovarian cysts, ovaries were dissected. Livers were preserved to evaluate EESA’s antioxidant properties. Histopathology analysis revealed that EESA reduced body weight and the number of cystic follicles. Furthermore, it also lowered the elevated levels of serum testosterone, luteinizing hormone, insulin, and malonaldehyde in PCOS rats while increasing the levels of follicle-stimulating hormone, estradiol, progesterone, prolactin, and other antioxidant enzymes such as superoxide dismutase, glutathione, and catalase. It can be concluded that EESA exhibited beneficial effects in normalizing the perturbed hormonal profile and improved the ovary status by decreasing the cystic follicle and improving the ovulation status in a dose-dependent manner.

## 1 Introduction

Polycystic ovarian syndrome (PCOS) is a complex reproductive illness characterized by multiple endocrine abnormalities that primarily affects females of reproductive age. PCOS symptoms include hyperandrogenism, hirsutism, painful and irregular menstrual periods, amenorrhea, an increased number of ovarian cysts, and anovulation. Infertility is commonly caused by PCOS.^1,2^ The disease prevalence ranges from 4 to 26% in different populations and regions of the world.^1,3,4^

Pathologically, PCOS is due to the distorted intracellular signaling which can arise either from extra- or intraovarian factors stimulating the synthesis of androgen from theca cells (endocrine cells in ovaries). The extra-ovarian factors include increased luteinizing hormone (LH), increased insulin level, or/and decreased follicle-stimulating hormone (FSH).^5,6^ The elevated LH levels can directly enhance the theca androgen biosynthesis via decreased mitogen activated protein kinase 1 (MAPK1).^6,7^ In addition, an increased level of insulin tends to interact with increased LH levels, which intensifies the intrinsic steroidogenic defect, leading to increased phosphoinositide 3 kinase (PI3K) and inositolphosphoglycan (IPG) activity, which in turn stimulates androgen production from theca cells.^8^ The lower FSH levels (in comparison to those of LH) may play an indirect role in androgen biosynthesis. Reduced FSH levels inhibit aromatase activity, resulting in less androgen-to-estrogen conversion and increased androgen levels in the ovaries, resulting in increased androgen biosynthesis.^5^

**Figure 1:**
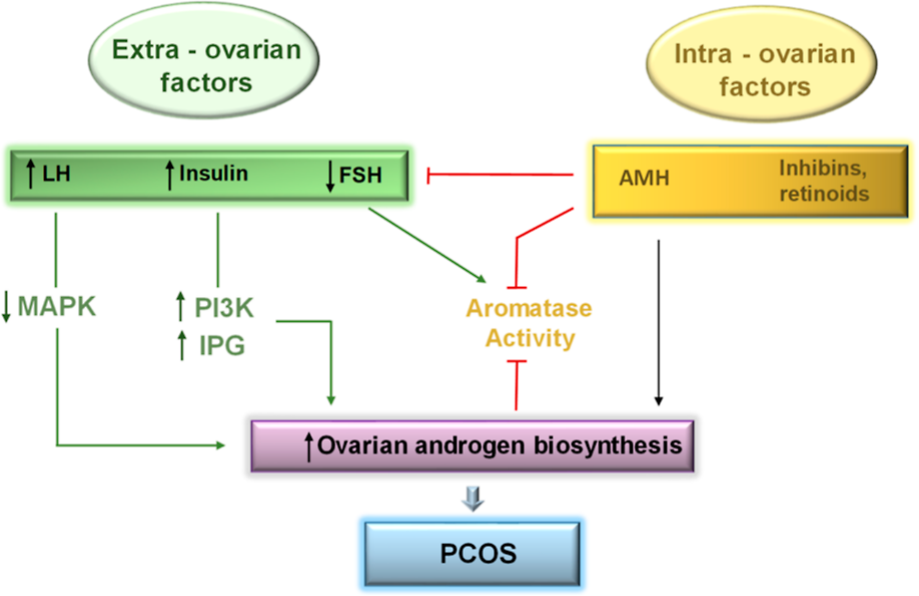
Pathogenesis of PCOS; hormone regulators and intracellular signaling defects causing excessive ovarian androgen production further leading toward PCOS; red arrows show inhibitory effect while green arrows show stimulating effect. Abbreviations: LH = luteinizing, FSH = follicle stimulating hormone, AMH = anti-Müllerian hormone, MAPK = mitogen activated protein kinase, PI3K = phosphoinositide 3 kinase, and IPG = inositolphosphoglycan.

The intraovarian factors include increased levels of anti-Müllerian hormone (AMH) and inhibins. The increased AMH action will reduce the aromatase activity in synergism with FSH.^9^ AMH and inhibins can play this role either directly via acting on theca cells to enhance the androgen synthesis or indirectly via suppression of FSH. Reduced aromatase activity causes hindrance in the transformation of androgen to estrogen, contributing to androgen excess in the ovaries. Consequently, elevated levels of androgen continue to ensure the suppression of aromatase activity.^5^Figure 1 explains the pathogenesis of PCOS. Early diagnosis and therapeutic intervention are crucial because the disease has been linked to a number of chronic conditions and comorbidities.^10^ In addition to a lack of knowledge about the disease, its management guidelines, and therapeutic approaches, women, particularly those from rural regions, are reluctant to visit gynecologists or endocrinologists for treatment while exhibiting signs of the condition. Most women do not receive treatment as a result of this resistance, which could have a number of negative effects in the future.^11^

There is currently no universal treatment for PCOS. As a result, treatment is always personalized and modified to meet the specific needs of each patient. Treatment is symptom-focused and may not be necessary.^12^ Lifestyle modifications and dietary and exercise interventions are adopted, especially for PCOS patients who are obese. However, significant clinical symptoms, including acne, hirsutism, irregular periods, and anovulation, necessitate pharmaceutical intervention. These include progestin-only and combination oral contraceptives (COCPs), along with metformin, spironolactone, and letrozole or clomiphene, all of which are used to treat infertility. By contrast, drugs may only be considered in women who are not trying to conceive.^13^ In patients having trouble in losing their weight, the usage of metformin is crucial for improving menstrual cycle management, ovulation rate, and pregnancy chances.^14^ Cosmetic procedures, including waxing, bleaching, and topically employed cream eflornithine (13.9%), can be applied immediately.^15^ COCPs normalize menstruation, raise sex hormone-binding globulin levels, lower androgen levels, avoid pregnancy, and provide protection to the endometrium. They raise total cholesterol, HDL, and TG levels in adult women; however, the data available do not indicate any substantial alterations in body weight or glucose tolerance.^16^

Existing approaches for treating PCOS are constrained by the prevalence of contraindications in PCOS women, medication failure, and potential for serious adverse effects.^17^ Nowadays, ayurvedic treatment is a popular choice because of the challenging downside of conventional treatments for PCOS in women.^18^ Women’s acceptance and use of complementary medicine have increased from 26 to 91% in the last ten years.^19^ Traditional herbal remedies are getting a lot of attention in global health discussions right now. In most recent times, the role of certain medicinal plants emerged to control multiple ailments, such as *Fagonia indica*, *Momordica charantia* L., *Curcuma longa*, *Cicer arietinum* L., *Nigella sativa*, *Phyllanthus muellerianus*, *Ocimum kilimandscharicum* L., *Cinnamomum verum*, *Ecklonia cava*, *Glycyrrhiza glabra*, *Trigonella foenum-graecum*, *Vitex negundo* L., *Allium fistulosum*, *Citrullus colocynthis*, *Linum usitatissimum*, *Corylus avellane*, and *Melilotus indicus* (L.).^4,20,21^

*Saraca asoca* (Roxb.) Willd. is a plant belonging to the family Fabaceae and the subfamily Caesalpiniaceae. It is commonly known as Ashoka. It has been used traditionally in the Indian system of treatment for the cure of genital, uterine, and other reproductive conditions in women, such as pain, fever, and inflammation.^22^*S. asoca* ethanolic extract reduces lipid alteration, decreases renal oxidative stress, and undeniably provides hypoglycemic, hypolipidemic, and antioxidant effects.^23^ The plant further possesses anti-inflammatory,^24^ antimenorrhagic,^25^ CNS depressant,^26^ analgesic,^26^ anthelmintic,^27,28^ antiulcer,^29^ antimutagenic,^30^ and antioxytocic activity.^28^ The present study aimed to evaluate the potential antiandrogenic activity of *S. asoca* in letrozole-induced PCOS-affected adult female rats and to provide a scientific rationale for the traditional uses of *S. asoca* (Roxb.) Willd. in hormonal disorders.

## 2 Material and Methods

### 2.1 Medicinal Plant Collection and Its Authentication

*S. asoca* (Roxb.) Willd. was obtained from Pakistan herb store, Peshawar. The plant specimen was collected in December 2021. It was then identified and verified by botanist Dr. Mansoor Ahmad, chairperson at the University of Agriculture, Faisalabad. Moreover, it was submitted to the herbarium of the University of Agriculture Faisalabad, Department of Botany, as a specimen with voucher number 142-01-2022 for future reference.

### 2.2 Plant Extract Preparation

The bark was first dried at shade, ground into a moderately fine powder using a mixer grinder and was extracted with ethanol. A simple maceration method was used for the extraction process. The dried powdered bark (1 kg) was allowed to soak at room temperature in sufficient quantity of ethanol (95%) and in a dark place. It is kept for 7 days with vigorous shaking 4–5 times daily. The solvent was filtered with the help of Whatman filter paper and then allowed to evaporate ethanol until dried in a rotary evaporator. After completing this procedure, a semisolid material (extract) was produced, which was then air-dried. Finally, 80 g of powdered *S. asoca* (Roxb.) Willd. Ethanolic extract (EESA) was obtained, and its percentage yield was 8%. The extract was preserved at 4 °C in a glass container, which was later administered to rats that had previously been induced with PCOS to investigate its therapeutic effects.^6^

### 2.3 Plant Characterization

To assess the phenolic and flavonoid content of *S. asoca* (Roxb.) Willd. ethanolic extract (EESA), HPLC was used. Sample of EESA was run in HPLC [model = 1269 infinity II, Quaternary pump, VWD (Variable Wavelength Detector), degreaser 1200 series, column Zorbex SB Eclipse-C18 mm, 5gm, Agilent company, USA], and several peaks on the chromatogram were obtained.

### 2.4 Total Flavonoid Content

After allowing the extract to stand for 5 min at room temperature, 0.6 mL of 10% AICl_3_ was added in it. Once more, the mixture was left at room temperature for 5 min. After waiting for 5 min, added NaOH (2 mL, 1 M) to it, and the volume was filled using distilled water. A spectrophotometer for the measurement of absorbance at 510 nm was used. Total flavonoid levels were determined using the calibration curve for catechin, which was utilized as a standard (at 10–130 ppm concentration). Total flavonoid content (TFC) was evaluated as the equivalent of catechin (CE).^31^
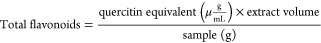
1

### 2.5 Total Phenolic Content

0.5 mL each of Folin–Ciocalteu reagent and extract solution (0.05 g/5 mL) were mixed. Subsequently, 7.5 mL of deionized water was added. After this, the solution had been allowed to stand at 25 °C for 10 min, and sodium carbonate (1.5 mL, 20% w/v) was added. After that, the mixture was allowed to be heated for 20 min in a water bath at 45 °C before being chilled in an ice bath. Finally, a spectrophotometer was used to measure the absorbance at a wavelength of 755 nm. The standard curve of gallic acid was used to quantify the total phenolic contents (TPC). Gallic acid was utilized as the standard (100–1300 ppm).^4^ Gallic acid equivalent (GAE) was used to assess TPC, and the results were represented as milligrams per gram of dry matter by using the following equation
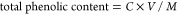
2*C* = concentration of gallic acid. *V* = volume of the extract solution in mL. *M* = weight of the extract in g.

### 2.6 DPPH (2,2-Diphenylpicrylhydrazyl) Assay

The practical solutions of standard “ascorbic acid” and those of the plant extracts (20, 40, 60, 80, and 100 μg/mL) were diluted in methanol for the experiment. The concentration of DPPH was maintained (2 mL, 0.004%). Varied concentrations of plant extract and standard were added. After being thoroughly mixed, the mixture was allowed to stand at room temperature and in the dark for 30 min. The mixture’s absorbance at 515 nm was measured spectrophotometrically. DPPH radical without the antioxidant, that is, blank’s absorbance was also measured. The following equation was used to measure the tendency to scavenge DPPH radicles.^32^
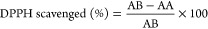
3AB: absorbance of blank at *t* = 0 min. AA: absorbance of an antioxidant (tested sample) at 30 min.

### 2.7 Animals Housing

Thirty female adult albino rats were acquired from the animal house of Government College University, Faisalabad, and retained in stainless-steel cages (5 rats/cage). They were kept for 2 weeks for the purpose of acclimatization. A pathogen-free barrier facility was provided to the animals. All animals were maintained at a controlled environment of 20 ± 2 °C temperature, relative humidity of 55 ± 5%, and a 12 h light/dark cycle and were fed with the standard diet and water was provided ad libitum. The guidelines by “US National Institutes of Health Guide for the Care and Use of Laboratory Animals” given in NIH edition no. 8, updated in 2011, were followed in conducting the study and were approved by the Government College University of Faisalabad’s Ethics Review Committee (ERC) under the ERC number of “GCUF/ERC/51”.

### 2.8 Induction of Disease

After rats were adapted toward the laboratory conditions, they were randomly divided into two pretreatment groups, that is, the control group (*n* = 5) and the PCOS group (*n* = 25). Control group was administered with 1 mL of 0.5% CMC, while the suspension of letrozole (1 mg/kg) in 0.5% carboxymethyl cellulose was administered p.o. to the ones in PCOS group for 7 weeks to induce PCOS in the female rats. This dose was selected in accordance with established protocols.^6^ During this time, the relative number of cornified cells, epithelial cells, and leukocytes seen under the light microscope was used to determine the estrous cycle every day, and weekly weight changes were noted as well.

### 2.9 Experimental Design and Sample Collection

All 30 animals were divided into 6 groups (*n* = 5) by a completely randomized approach. Groups were designated as normal control, disease/PCOS control, metformin, EESA 200 mg/kg, EESA 400 mg/kg, and EESA 600 mg/kg (Table 1). On their tails, they were marked with permanent markers of various colors to help identify them. Each dose was administered orally over the course of 7 weeks using gavage and the daily examination of the vaginal smear under Accu-Scope. After 5 weeks of treatment, all animals was weighed and were euthanized after a few hours of receiving the last dose. Cardiac puncture was done to obtain a blood sample, and then was centrifuged to separate the serum (15 min at 3000 rpm) and frozen at −20 °C for hormonal and biochemical analysis. Ovaries were removed and were dissected for the purpose of histopathological analysis. Livers were also preserved for the purpose of evaluating their antioxidant potential.

**Table 1:** Dosage Protocol for Experimental Design^a^

disease induction phase duration: 7 weeks (week 1–7) route: p.o.			treatment phase duration: 5 weeks (week 8–12) route: p.o.		
group name	Formulation	dose	group name	formulation	dose
normal control group (*n* = 5)	0.5% w/v CMC	10 mL/kg	normal control group (*n* = 5)	0.5% w/v CMC	10 mL/kg
			disease/PCOS group (*n* = 5)	letrozole in 0.5% w/v CMC	1 mg/kg/day
letrozole-induced PCOS groups.(*n* = 25)	letrozole in 0.5% w/v CMC	1 mg/kg/day	standard treatment (metformin) group (*n* = 5)	metformin in 0.5% w/v CMC	20 mg/kg/day
			ethanolic extract of S. asoca (EESA) treatment group (*n* = 5)	S. asoca in 0.5% w/v CMC	200 mg/kg/day
			ethanolic extract of S. asoca (EESA) treatment group (*n* = 5)	S. asoca in 0.5% w/v CMC	400 mg/kg/day
			ethanolic extract of S. asoca (EESA) treatment group (*n* = 5)	S. asoca in 0.5% w/v CMC	600 mg/kg/day

a CMC: carboxymethylcellulose.

### 2.10 Monitoring of Estrous Cycle and Cytology of Vaginal Smear

The estrous cycle has four phases, including pro-estrus, estrus, met-estrus, and diestrus. The already stated method was used to determine these estrous cycle stages. Nucleated epithelial cells predominate in the proestrus stage, with a limited proportion of leukocytes. Cornified epithelial cells are the main component of the estrus phase. Leukocytes-nucleated cornified cells and cornified epithelial cells are all present throughout the met-estrus phases; however, only leukocytes predominate during the diestrus stage, along with increased mucus. Using a sterilized cotton swab, the various estrous cycle stages were assessed. After being soaked in 0.9% normal saline, the cotton swab was inserted into the rat’s vagina. Mucus from the vagina was then spread out on a glass slide. After the mucus on the slide had nearly dried, it was stained with methylene blue dye and allowed to dry for another 2 min at room temperature. The cytology of the vagina was assessed using a light microscope with a camera (Accu-Scope).^6^

### 2.11 Histopathology of Rats’ Ovaries

Rat ovaries were first removed during dissection and fixed by immersing them in 10% formalin. The ovarian tissues were correctly trimmed to provide an appropriate size and orientation. The tissue sample was then pre-embedded with paraffin wax of histology grade. After paraffin had penetrated the tissue, it was gently removed from cassettes and inserted into the mold. The ovarian tissues were cut into thin (4–5 mm thick) pieces from paraffin blocks. Precision knives were used to cut tissue into sections (microtomes). The histological alterations in ovarian tissue were evaluated using the hematoxylin and eosin (H&E) stain. After sectioning, paraffin blocks were stored at room temperature for later use. Additionally, stained slides were kept in the proper container to prevent prolonged exposure to light. Following slide preparation, a compound microscope was used to photograph these slides (Accu-Scope).^31^

### 2.12 Hormone Analysis

Enzyme-linked immunosorbent assay (ELISA) kit and radio immunoassay (RIA) techniques were used to analyze the serum hormones. A heart puncture was used to get blood samples, and then cold centrifugation was used to extract the serum. RIA (Gamma counter) was used to assess the levels of serum FSH (mIU/mL), progesterone (ng/mL), prolactin (ng/mL), and testosterone (ng/dL) using a kit from Beckman Coulter, Inc., USA. While serum levels of LH (mL/mL) were measured using an ELISA kit from Pointe Scientific Inc., US. In addition, serum estrogen concentration (pg/mL) was measured using a kit from ALPCO, USA, and insulin concentration (μIU/mL) within the serum was measured using a kit from Cal biotech, USA.

### 2.13 Liver Function Tests

Various markers of liver functioning, that is, levels of total bilirubin, alanine transaminase (ALT), aspartate aminotransferase (AST), alkaline phosphatase (ALP), and albumin, were evaluated using a semiautomated chemical analyzer to perform a spectrophotometric liver functional test (Microlab-300).

### 2.14 Antioxidant Enzyme Assay

Antioxidant enzyme levels, such as superoxide dismutase (SOD), glutathione (GSH), and catalase (CAT), are frequently found to be low in PCOS along with raised malondialdehyde (MDA) levels. Therefore, the level of these enzymes was estimated during the study period. Liver homogenates were prepared with the help of a tissue homogenizer and phosphate buffer 0.1 M (pH 7.4), which is made up of potassium chloride (10 mM), ethylene diamine tetra-acetic acid (1 mmol), sucrose (0.25 M), and phenylmethylsulfonylfluoride (1 mM). The mixture was allowed to centrifuge at 800 rpm for 30 min at 4 °C to get the supernatant. Afterward, evaluation of antioxidant parameters (CAT, MDA, SOD, and GSH) was done using this supernatant.^21^

### 2.15 Statistical Analysis

Results are denoted as mean ± SEM. GraphPad Prism (version 8.4.3) was used to conduct the statistical analysis. Analysis of variance (ANOVA) was used for multiple comparisons, followed by Tukey’s test. The findings were regarded as statistically significant if *p* < 0.05.

## 3 Results

### 3.1 Characterization of Plant

**Figure 2:**
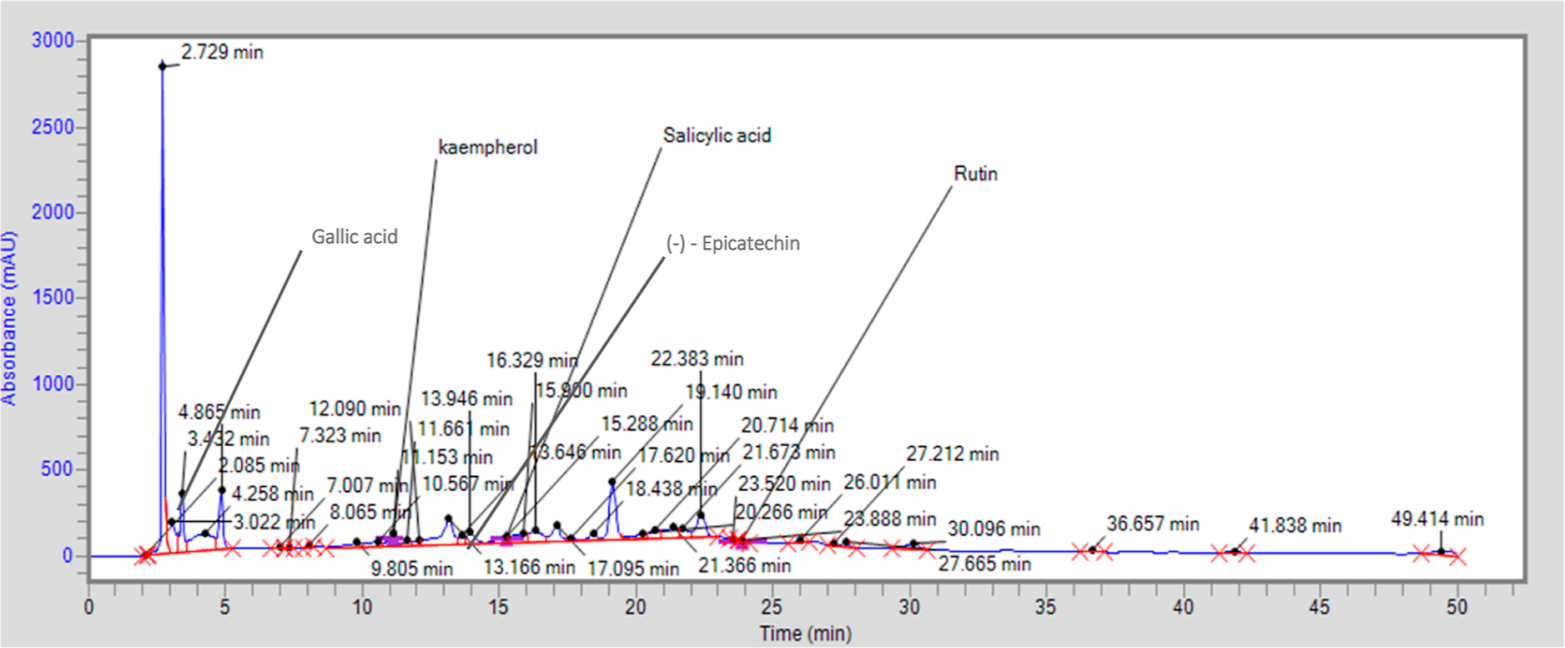
HPLC of the ethanolic extract of *S. asoca* (Roxb.) Willd.

**Figure 3:**
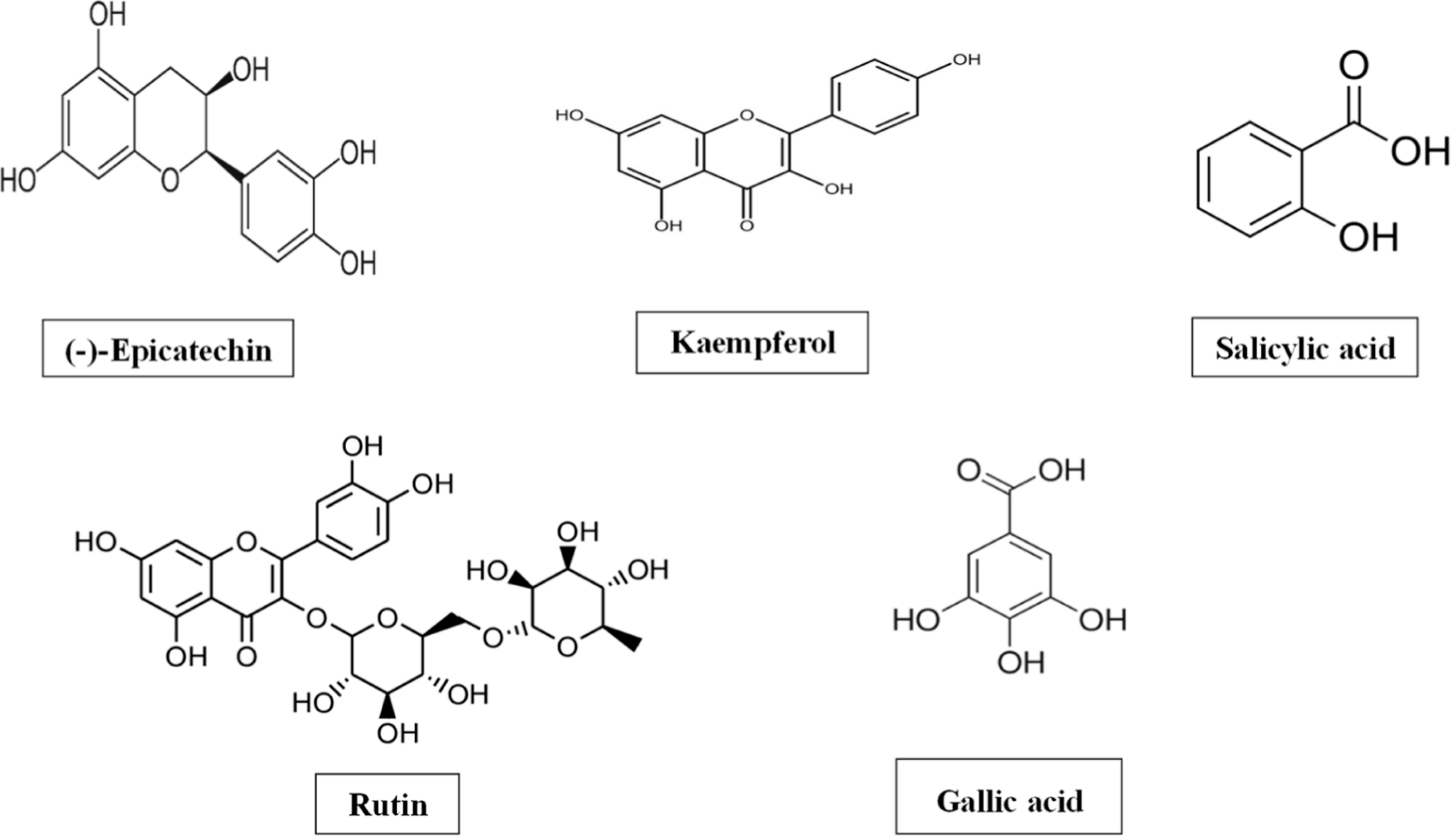
Chemical structures of various phyto-constituents detected in *S. asoca* (Roxb.) Willd. Ethanolic extract through HPLC.

HPLC of plant extract was done (Figure 2), and the flavonoid contents detected in the ethanolic extract of *S. asoca* were Kaempferol (4.178 mg/kg), Rutin (1.119 mg/kg), and (−)-Epicatechin (5.719 mg/kg). Moreover, the phenolic contents found were salicylic acid (4.258 mg/kg) and gallic acid (2.880 mg/kg). Structures of these constituents are listed in Figure 3. The TFC of EESA, given as the catechin equivalent (CE), was found to be 12.21 ± 0.18. The TPC in EESA, given as gallic acid equivalent (GAE), was found to be 97.86 ± 0.84.

Percent inhibition of the radical DPPH by *S. asoca* ethanolic extract is mentioned in Table S1 and Figure S1. Table S1 and Figure S1 are given in the Supporting Information for publication. Sample was taken in μg/mL. To compare the results, ascorbic acid was selected as a reference. The IC_50_ value of *S. asoca* was 115.2 μg/mL as compared to that of ascorbic acid, of which the IC_50_ value was 48.21 μg/mL.

### 3.2 Body Weight Changes

**Figure 4:**
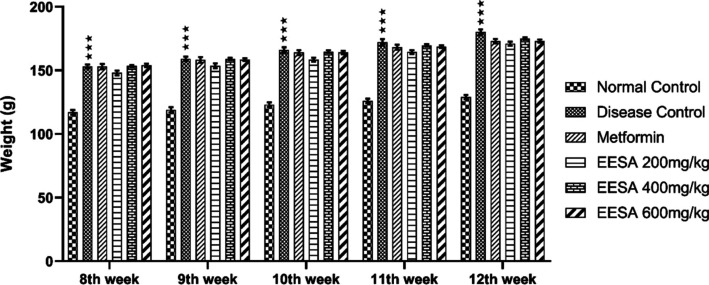
Effect of we asoca Willd. (ethanolic extract) EESA on body weight (g) in PCOS rat model induced by letrozole. Data are presented as mean ± SEM (*n* = 5). Groups were compared by two-way analysis of variance (ANOVA), followed by Tukey’s multiple comparison test. *p < 0.05; **p < 0.01; and ***p < 0.00. *Depicting statistically significant difference from the normal control.

During disease induction, animals who received letrozole had significantly (*P* < 0.05) higher body weight, that is, 34.5%, than the control group, that is, 20%. This almost one and a half times increased body weight of PCOS rats indicated the induction of disease in them. At the end of the trial period, rats from the disease group weighed more (17.64%) than rats from the normal control group (10.25%). When compared to the disease group, the weight gain in all treatment groups, that is, metformin (13.05%), EESA 200 mg/kg (15.54%), 400 mg/kg (14.08%), and 600 mg/kg (12.33%) was lower as compared to the disease group showing the therapeutic potential of EESA in weight reduction (Figure 4).

### 3.3 Estrus Cycle Monitoring and Vaginal Smear Cytology

**Figure 5:**
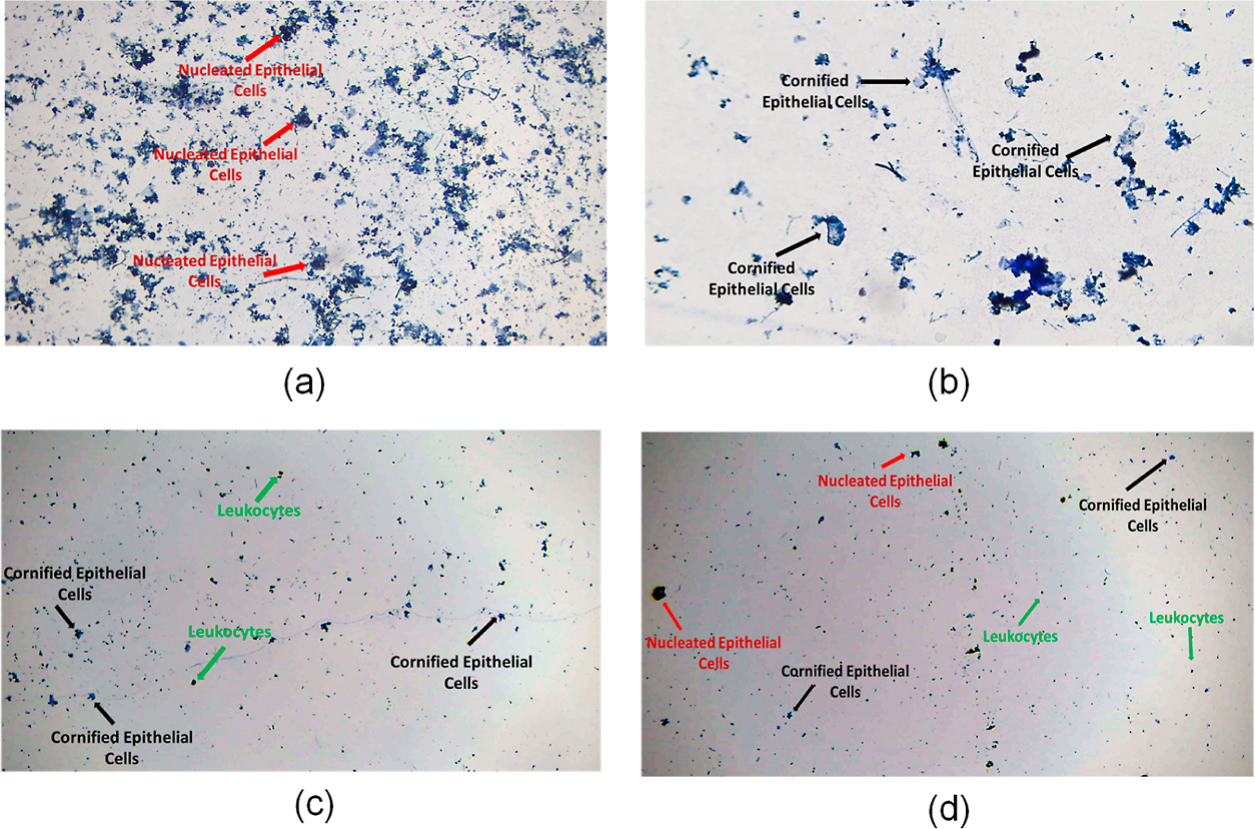
Vaginal smear cytology of the normal control group’s rats depicting various phases of the estrous cycle: (a) pro-estrous phase contains mostly nucleated epithelial cells (red arrow). (b) Estrous phase is comprised of cornified epithelial cells (black arrow). (c) Met-estrous phase comprises mostly of leukocytes (green arrow) (d) diestrous phase contains all nucleated epithelial cells, cornified epithelial cells, and leukocytes.

**Figure 6:**
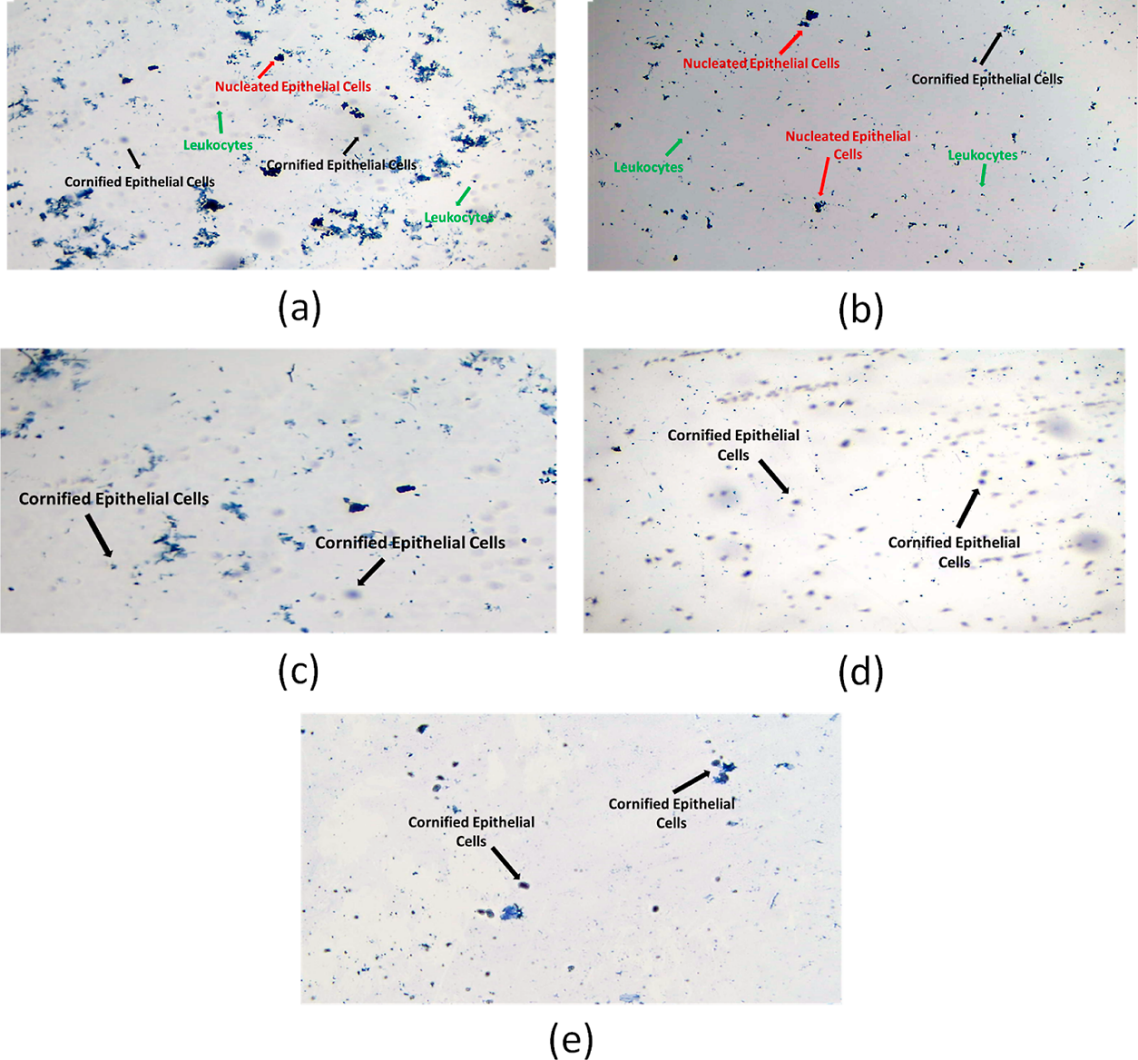
Vaginal smear cytology of PCOS and treatment group’s rats depicting their estrus phase: (a) group with PCOS in the diesteric phase. (b) Group treated with metformin in the diesteric phase. (c) Group treated with EESA 200 mg/kg in the estrous phase. (d) Group treated with EESA 400 mg/kg in the estrous phase. (e) Group treated with EESA 600 mg/kg in the estrous phase of the cycle.

Throughout the entire study, different phases of the estrus cycle (i.e., pro-estrous, estrous, met-estrus, and diesterrus) occurred at regular intervals in normal control rats, depicting a typical estrus cycle as shown in Figure 5. Whereas animals in the PCOS group had a delayed estrous cycle and spent more time in the diestrus stage, since the vaginal smear contained all types of cells that is cornified epithelial cells, leukocytes, and nucleated epithelial cells and which are indicative of the diestrus phase. Standard group, which received metformin treatment, was also in the diestrus phase. While the estrous phase of the cycle, which is primarily made up of cornified epithelial cells, was evident in all *S. asoca* treatment (200, 400, and 600 mg/kg) groups (Figure 6).

### 3.4 Effect of Ethanolic Extract of *S. asoca* (Roxb.) Willd. on Histopathology of Rat Ovaries

**Figure 7:**
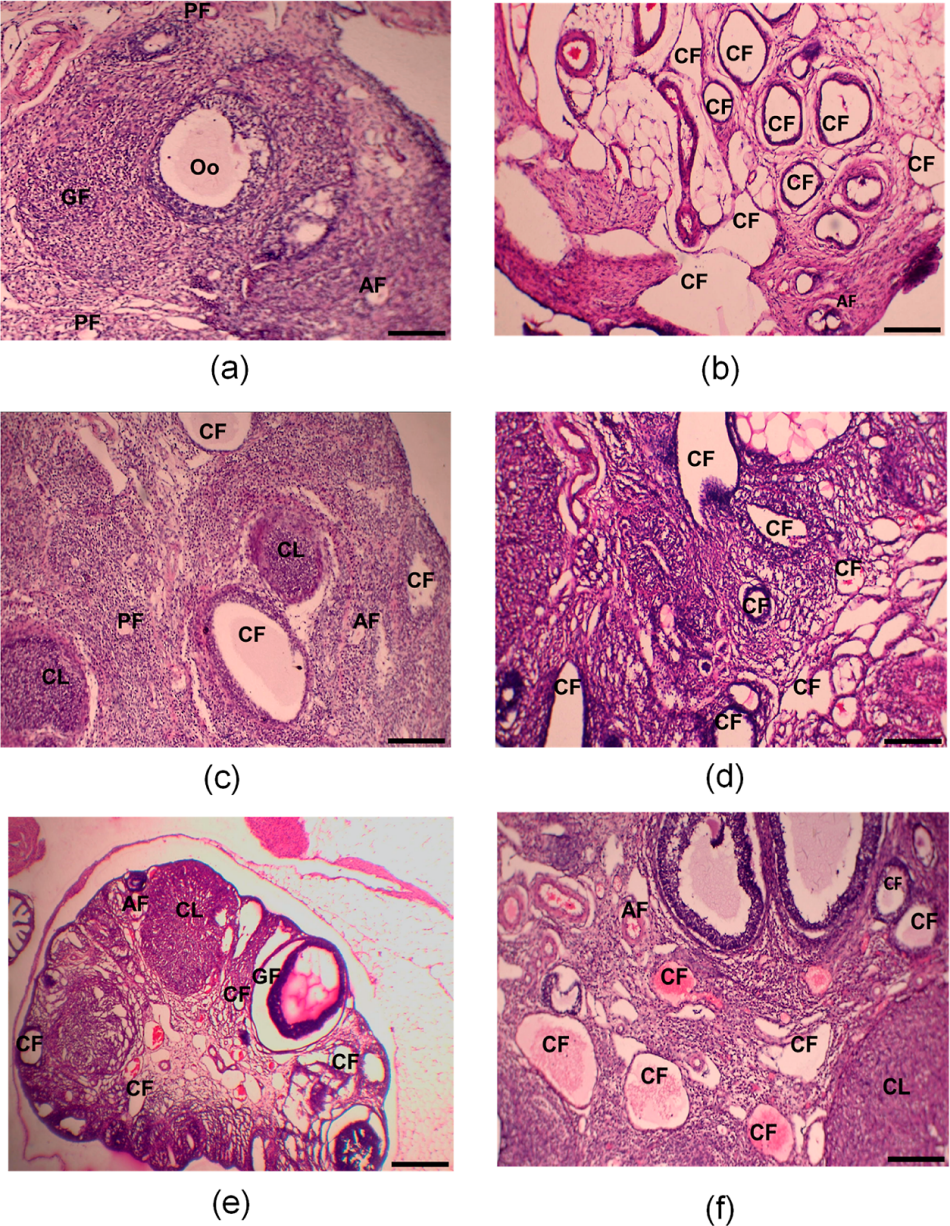
Effect of the ethanolic extract of *S. asoca* (Roxb.) Willd. (EESA) on ovarian histopathology, H&E-stained cross section of ovaries (4× magnification), (a) section from the normal control group displaying normal morphology of ovarian follicles in various stages. (b) PCOS rat ovary comprising numerous cystic follicles. (c) PCOS rat ovary treated with metformin (20 mg/kg) containing reduced cystic follicles. (d) PCOS rat ovary treated with EESA, 200 mg/kg. (e) PCOS rat ovary treated with EESA 400 mg/kg. (f) PCOS rat ovary treated with EESA 600 mg/kg. Abbreviations represent: CL: corpus luteum, CF: cystic follicles, AF: atretic follicles, DF: developing follicles, PF: primary follicles, Oo: oocyte. Scale bar = 100 μM.

The ovarian histopathological slides of normal control groups showed typical morphology, including tiny to medium-sized antral follicles, corpus luteum, oocytes, and follicles in various phases of development, that is, primary follicles, growing follicles, and so forth (Figure 7a). The disease control group’s ovarian slides showed atretic antral follicles, many cystic follicles, and disordered granulosa cell compartments with variable granulosa cell thickness. They also lacked corpus luteum (Figure 7b). These alterations in this group can be attributed to lower FSH and elevated testosterone levels. However, administration of metformin (20 mg/kg) and ethanolic extract of *S. asoca* (200 and 400 mg/kg) resulted in a decreased number of cystic follicles and a rising number of developing follicles and corpus luteum (Figure 7c–e). While ovaries of rats in the group receiving EESA 600 mg/kg significantly restored their normal histology (Figure 7f).

### 3.5 Effect of Ethanolic Extract of *S. asoca* (Roxb.) Willd. (EESA) on Hormone Level

**Figure 8:**
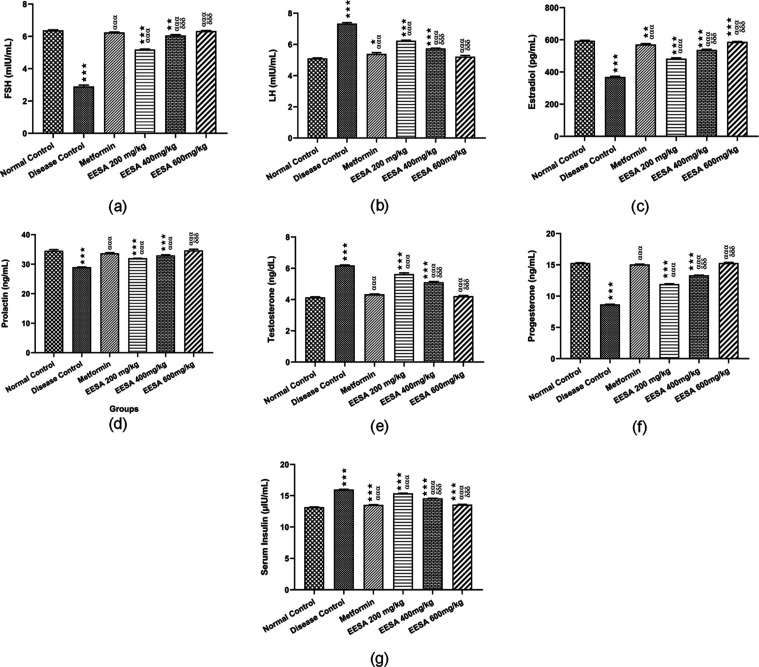
Effect of *S. asoca* (Roxb.) Willd. (ethanolic extract) EESA on hormones (a) FSH (b) LH (c) estradiol (d) prolactin (e) testosterone (f) progesterone and (g) serum insulin in PCOS rat model induced by letrozole. Data is presented as mean ± SEM (*n* = 5). Groups were compared by two-way analysis of variance (ANOVA), followed by Tukey’s multiple comparison test. *^αδ^*p* < 0.05; **^ααδδ^*p* < 0.01; and ***^αααδδδ^*p* < 0.001. *Depicting statistically significant difference from the normal control.^α^Depicting statistically significant difference from the disease group. ^δ^Depicting statistically significant difference from EESA 200 mg/kg.

In the disease group, a significant (*p* < 0.001) reduction in serum FSH level was seen upon comparison with the normal control group. However, its level significantly elevated in all the treatment groups; metformin, EESA 200, 400, and 600 mg/kg groups when compared with the disease group. The most significant (*p* < 0.001) effects were observed in the EESA 600 mg/kg group (Figure 8a). A significant (*p* < 0.001) elevation in serum LH and testosterone levels was seen in disease group II (PCOS) when compared with the normal control group, while the level of both hormones significantly decreased in all treatment groups; metformin, EESA 200, 400, and 600 mg/kg groups when compared with the disease group. If all the doses of EESA were compared, the most significant decremental effects were observed with EESA 600 mg/kg (Figure 8b,c).

A significant (*p* < 0.001) reduction in serum levels of estradiol and prolactin was observed in the disease group when compared with the normal control group and their level significantly elevated in all treatment groups; metformin, EESA 200, 400, and 600 mg/kg groups, and the most significant (*p* < 0.001) effects were observed in the EESA 600 mg/kg group (Figure 8d,e). A significant (*p* < 0.001) reduction in serum progesterone level was observed in the disease group upon comparison with the normal control group. Moreover, the level significantly elevated in all treatment groups; metformin, EESA 200, 400, and 600 mg/kg (Figure 8f). In addition, a significant (*p* < 0.001) elevation in serum insulin level was also observed in the disease group when compared with the normal control group. However, its level significantly decreased in all treatment groups; metformin, EESA 200, 400, and 600 mg/kg groups upon comparing with the disease group (Figure 8g).

### 3.6 Effect of Ethanolic Extract of *S. asoca* (Roxb.) Willd. on Liver Functioning Test

A statistical difference was seen in the concentration of total bilirubin in the disease group from the normal control group, as well as in all the treatment groups; metformin, EESA 200, 400, and 600 mg/kg groups from the disease group. AST and ALP levels reduced significantly within the disease group when compared with the normal control group. There was also a significant difference in the concentration of AST and ALP in metformin, EESA 200, 400, and 600 mg/kg groups upon comparison with the disease group. Level of alanine transferase (ALT) reduced nonsignificantly in the disease group from the control group, while a significant difference was seen within metformin, EESA 200, 400, and 600 mg/kg groups upon comparison with the disease group. There was also a significant difference in the value of albumin between the disease and normal control group as well as when all treatment groups were compared with the disease group (Table 2).

**Table 2:** Effect of *S. asoca* (Ethanolic Extract) on (Roxb.) Willd. on Liver Function Parameters; Total Bilirubin, ALT, AST, ALP, Albumin^a^

parameters	group I (normal control)	group II (disease control)	group III (metformin)	group IV (EESA 200 mg/kg)	group V (EESA 400 mg/kg)	group VI (EESA 600 mg/kg)
total bilirubin (mg/dL)	0.5320 ± 0.028	0.4380 ± 0.035***	0.5160 ± 0.024^ααα^	0.5140 ± 0.016^αα^	0.4300 ± 0.018 ***	0.5320 ± 0.023 ^ααα^
ALT (U/L)	78.66 ± 0.237	76.96 ± 0.120	72.84 ± 0.508***^α^	91.36 ± 1.000***^ααα^	143.5 ± 1.352***^ααα^	88.02 ± 0.858***^ααα^
AST (U/L)	442.3 ± 1.617	408.9 ± 2.405***	444.0 ± 2.529^ααα^	478.3 ± 2.804***^ααα^	576.0 ± 1.776***^ααα^	642.0 ± 2.061***^ααα^
alkaline phosphate (U/L)	817.0 ± 3.289	790.5 ± 2.951***	508.2 ± 2.220***^ααα^	846.7 ± 3.378***^ααα^	991.5 ± 2.401***^ααα^	630.4 ± 2.261***^ααα^
albumin (g/dL)	3.232 ± 0.020	4.064 ± 0.018***	4.160 ± 0.017a***^α^	4.272 ± 0.020***^ααα^	3.918 ± 0.018***^ααα^	4.148 ± 0.014***^α^

a Data is presented as mean ± SEM (*n* = 5). Groups were compared by two-way analysis of variance (ANOVA), followed by Tukey’s multiple comparison test. *^α^*p* < 0.05; **^αα^*p* < 0.01; and ***^ααα^*p* < 0.001. *Depicting statistically significant difference from the normal control.^α^Depicting statistically significant difference from the disease group. ALT: alanine transaminase; AST: aspartate aminotransferase.

### 3.7 Effect of Ethanolic Extract of *S. asoca* (Roxb.) Willd. (EESA) on Antioxidant Enzymes

A significant (*p* < 0.001) reduction in hepatic enzymes SOD, catalase peroxidase (CAT) and GSH was found in the disease group when compared with the normal control group, which demonstrated that letrozole increased oxidative stress and decreased antioxidant enzyme levels. While the level of these enzymes significantly elevated in all treatment groups; metformin, EESA 200, 400, and 600 mg/kg groups when compared with the disease group. Moreover, a significant (*p* < 0.001) elevation in the hepatic enzyme MDA was seen in the disease group when compared with the normal control group, while its level significantly reduced in treatment groups; metformin, EESA 200, 400, and 600 mg/kg groups when compared with the disease group. Table 3 illustrates how these antioxidant indicators changed throughout time.

**Table 3:** Effect of *S. asoca* (Roxb.) Willd. (Ethanolic Extract) EESA on Antioxidant Enzymes (a) SOD, (b) CAT, (c) GSH, and (d) MDA in the PCOS Rat Model Induced by Letrozole^a^

parameters	group I (normal control)	group II (disease control)	group III (metformin)	group IV (EESA 200 mg/kg)	group V (EESA 400 mg/kg)	group VI (EESA 600 mg/kg)
SOD (IU/μL)	0.0662 ± 0.003	0.0120 ± 0.007***	0.0632 ± 0.003^ααα^	0.0540 ± 0.005^ααα^	0.0626 ± 0.003^ααα^	0.0640 ± 0.005^ααα^
CAT (IU/μL)	0.8340 ± 0.025	0.3904 ± 0.049***	0.6958 ± 0.031*^ααα^	0.6600 ± 0.015**^ααα^	0.6952 ± 0.011**^ααα^	0.7244 ± 0.009^ααα^
GSH (U/mg)	0.7900 ± 0.020	0.2180 ± 0.025***	0.6880 ± 0.029 *^ααα^	0.4840 ± 0.020 ***^ααα^	0.5700 ± 0.015 ***^αααδ^	0.7080 ± 0.026^αααδδδ^
MDA (mg/mL)	70.63 ± 0.307	84.30 ± 0.308***	69.49 ± 0.344^ααα^	73.10 ± 0.279 ***^ααα^	59.69 ± 0.336 ***^αααδδδ^	55.45 ± 0.380***^αααδδδ^

a Data is presented as mean ± SEM (*n* = 5). Groups were compared by one-way analysis of variance (ANOVA), followed by Tukey’s multiple comparison test. *^αδ^*p* < 0.05; **^ααδδ^*p* < 0.01; and ***^αααδδδ^*p* < 0.001. *Depicting statistically significant difference from the normal control.^α^Depicting statistically significant difference from the disease group. ^δ^Depicting statistically significant difference from EESA 200 mg/kg.

## 4 Discussion

PCOS is a complex, polygenic illness having a significant economic and personal costs.^33^ Hyperandrogenism, anovulation, and follicular cysts are some of the clinical symptoms of PCOS that are linked to metabolic issues such as insulin sensitivity, hyperinsulinemia, dyslipidemia, and cardiovascular diseases.^1^ In addition to lowering level of androgens, weight of body and long-term risks for diabetes/cardiovascular disease, PCOS’s treatment also seeks to recover ovulation in women who wants to conceive.^34^ Letrozole, an aromatase (CYP450 enzyme) inhibitor, was employed to induce PCOS. Rats were given letrozole (1 mg/kg for 7 weeks) to induce PCOS. Regular vaginal checks showed the model’s effectiveness, and it was seen that letrozole groups had increased leukocyte counts, demonstrating a continuous diestrous phase. The letrozole prevents the change of androgens to estrogens, which causes hyperandrogenism, disrupts the estrous cycle, and increases the weight of the body and reproductive organs.^35^

In Ayurvedic medicine, the ethanolic extracts of *S. asoca* (Roxb.) Willd. (EESA) are said to be very effective for treating a variety of illnesses. Ashoka has long been recommended in Indian Ayurveda for irregular menstruation, especially in dysfunctional uterine bleeding (DUB) and as a uterine tonic.^36^ Here in this study, it was found that the body weight of the rats after PCOS-induction incsreded signifcantly, whereas all treatment groups’ body weight gain remained less over the period of 5 weeks. This body weight loss can be due to the presence of flavonoid constituents in EESA. EESA contains flavanoid rutin, another name for which is quercetin-3-*O*-rutinoside.^37^ Chronic Kaempferol treatment also reduced the number and ratio of activated microglia in the arcuate nucleus by 43 and 30%, respectively, followed by body weight loss, a decline in FE (feed efficiency), a drop in fasting blood glucose, and a propensity to enhance insulin sensitivity. The effects on energy balance (EB) brought on by peripheral treatment of kaempferol were finally replicated by its acute central administration. These findings imply that kaempferol may prevent obesity by controlling central mechanisms involved in the regulation of EB and inflammation of the hypothalamus.^38^

Moreover, the primary factor influencing ovarian physiology is the histology of the vaginal smear. In the disease control group, the vaginal smear analysis showed numerous leukocytes that indicated the diester stage of the estrous cycle, which is indicative of disease induction. The diester stage of PCOS rats continued for a long time. However, when EESA was used, it eliminated the estrous cycle irregularities and controlled the cyclicity. Histopathological slides of ovaries suggested that, unlike the normal control group, the disease group’s rats had acyclicity and ovarian cysts. These alterations are because in disease group rats there are higher testosterone levels and lower FSH levels. The histological slides of rats in the group receiving EESA 200 mg/kg showed a reduced cyst count and evidence of follicular development, demonstrating the positive effects of EESA, which raises FSH levels and lowers testosterone levels. The group receiving EESA 400 mg/kg further improved the ovarian cysts and the number of developing follicles. Furthermore, increasing the dose of EESA up to 600 mg/kg remarkably restored the normal physiology of ovaries. Thus, this confirms the dose-dependent beneficial effects of EESA.

After disease induction, there were elevated levels of serum LH, insulin, and testosterone in rats while lowering FSH, estradiol, prolactin, and progesterone levels. This hormonal change or illness state is because of disruption of the healthy hypothalamic-pituitary-gonadal axis, which results in elevated levels of both testosterone and LH.^6^ LH increases the level of testosterone release from ovarian theca cells by stimulating the enzyme 17-α hydroxylase, which stimulates the synthesis of testosterone from progesterone. Progesterone levels drop as a result, while androgen (testosterone) level rises.^1^ The coexistence of hyperandrogenism and a decline in FSH level is highly typical in the letrozole-induced PCOS model. People with PCOS may have higher levels of androgen and FSH receptors due to increased androgen levels,^39^ which would result in a negative feedback loop that would lower the blood level of FSH.^17^ In the present study, the antiandrogenic effects seen in treatment groups of ethanolic extract of *S. asoca* (EESA) may be associated with the presence of certain flavonoid compounds in it. Flavanols (myricetin, quercetin, fisetin, and kaempferol) are structurally related to estrogens, and because of this similarity, it was construed that they produce their effect either by directly affecting androgen levels or by competing with produced androgens for their receptor sites. These flavanols inhibit 5α-reductases, putatively decreasing the level of DHT.^40^ Also, kaempferol plays a role in androgenic signal mediation by targeting androgen receptor (AR) and consequent interference with androgen-induced effects. Rutin also possesses some levels of antiandrogenic and estrogenic effects comparable to those of metformin.^41^

The experimental results also revealed that the level of insulin was noticeably greater in PCOS rats in comparison with the normal control group. Hyperinsulinemia may increase the expression of the chemerin gene in polycystic ovaries, where chemerin may contribute to the pathogenesis of PCOS by directly acting on the ovary.^42^ However, there was a decrease in serum insulin levels in rats treated with the ethanolic extract of *S. asoca*. This decrease may occur for a variety of causes. One may be the presence of rutin, as it may cause reduction in the expression of the chemerin gene and an ultimate lowering of serum insulin level.^43^ Thus, our findings support that EESA produces beneficial and dose-dependent effects in lowering insulin resistance and altering serum insulin levels in PCOS rats.

Furthermore, PCOS-associated insulin resistance and hyperglycemia result in extreme lipid peroxidation, an increase in free radicals, and an exhaustion of antioxidants and are thereby related with induced oxidative stress in the reproductive tissue.^44^ Since lipid peroxidation results in free radical damage to the cell membrane components and induces inflammation and cell necrosis, it is frequently utilized as a marker for oxidative tissue damage.^45^ PCOS group in the present study had a significantly higher MDA level and lower SOD, CAT, and GSH activity. The flavonoid components (kaempferol, rutin, and (−)-epicatechin) in EESA may be responsible for the restoration of MDA, SOD, CAT, and GSH levels in groups that had received EESA, as the extract showed to have 73% radical scavenging activity at 100 μg/mL DPPH inhibition. As supported by Yang in the literature, substantial DPPH radical scavenging action was shown by rutin, which demonstrated an inhibition of 90.4% at a 0.05 mg/mL concentration. Rutin also demonstrated good lipid peroxidation inhibition.^46^ According to several studies, rutin considerably lessened the gastro mucosal damage caused by the necrotizing agent’s intragastric instillation and boosted GSH activity.^47^ A few researchers mixed rutin with additional antioxidants, which protected LDL in a synergistic manner against both lipid and protein oxidation.^48^ Another study claimed a kaempferol-induced increase in DPPH and ABTS (2,2′-azino-bis(3-ethylbenz-thiazoline-6-sulfonic acid)) radical scavenging ability, suppression of concanavalin A (Con A)-induced activation of T cell proliferation, and reduction of NO (nitric oxide) or ROS (reactive oxygen species) formation in LPS-induced RAW 264 (macrophage cell line).^49^ (−)-epicatechin somewhat also possesses antioxidant activity, as observed by Dueñas.^50^ It can also be hypothesized that the antioxidant activity of the ethanolic extract of *S. asoca* is because of the presence of several hydroxyl groups on the phenyl rings of rutin, (−)-epicatechin, and kaempferol.

Another way that reactive species can spread and cause harm is through lipid peroxidation. Lipid hydroperoxide levels in cellular and subcellular membranes increased because of an uncontrolled reaction. Gallic acid prevented the peroxidation of lipids due to its ability to scavenge free radicals and reduce lipid peroxidation.^4^ Also, salicylic acid treatment improved phenylalanine ammonia-lyase (PAL) and hydrophilic total antioxidant activity (H-TAA) and phenolic content in apricot fruit via controlling the metabolism of H_2_O_2_ during postharvest storage.^37^ Therefore, the antioxidant activity shown by *S. asoca* can also be attributed to the presence of phenolic constituents that is salicylic acid and gallic acid.

## 5 Conclusions and Future Recommendations

This study confirmed that the ethanolic extract of *S. asoca*, which had previously been used as a traditional remedy, can have therapeutic implications in alleviating PCOS symptoms by restoring body weight gain, hormonal profile, liver functioning, and antioxidant activity of enzymes in a dose-dependent manner. In addition, it reduced the number of cystic follicles in the ovaries. The effects of EESA were particularly dose-dependent, with the dose of 600 mg/kg having significantly greater ameliorative potential.

Nonetheless, the study was unable to establish a causal relationship between EESA and the aberrant endocrine-metabolic functions of letrozole-induced PCOS or to explain the molecular mechanism underlying EESA’s beneficial effects. However, it is possible to conclude that EESA has the potential to manage PCOS comorbidities in the long run. Furthermore, it ushers in a new era in which more molecular research can be conducted to determine the mechanism of action underlying this effect.

## Supporting Information Available

DPPH radical scavenging activity related data (PDF)
