# Supplementary material for: Phytochemical-Based Study of Ethanolic Extract of Saraca asoca in Letrozole-Induced Polycystic Ovarian Syndrome in Female Adult Rats
Source: ACS Omega. 2023 Nov 1;8(45):42586–97. doi: 10.1021/acsomega.3c05274 (PMC10652831; doi:10.1021/acsomega.3c05274)
Supplement: Supplementary file 1 — ao3c05274_si_001.pdf [file ao3c05274_si_001.pdf]

Phytochemicals Based Study of Ethanolic Extract of *Saraca asoca* in Letrozole Induced Polycystic Ovarian Syndrome in Female Adult Rats

Na Bu[1#], Alina Jamil[2#], Liaqat Hussain [2]*, Abdulrahman Alshammari [3], Thamer H. Albekairi[3], Metab Alharbi [3], Ayesha Jamshed [4], Rizwan Rashid Bazmi[5], Anam Younas[2]

[1]Department of Pharmacy, Women's Hospital, School of Medicine, Zhejiang University, Hangzhou, P.R. China, 31006.

[2]Department of Pharmacology, Faculty of Pharmaceutical Sciences, Government College University, Faisalabad, Pakistan.

[3]Department of Pharmacology and Toxicology, College of Pharmacy, King Saud University, Post Box 2455, Riyadh, 11451, Saudi Arabia.

[4]Department of Pharmacology, Faculty of Pharmacy, Islamia University Bahawalpur, Pakistan.

[5]Department of Pharmaceutical Chemistry, Faculty of Pharmaceutical Sciences, Government College University, Faisalabad, Pakistan.

[#] These authors contributed equally.

***Correspondence**

Dr. Liaqat Hussain
Ph.D.
Assistant Professor
Department of Pharmacology,
Faculty of Pharmaceutical Sciences,
Government College University Faisalabad, Pakistan.
liaqat.hussain@gcuf.edu.pk

1 DPPH radical scavenging Activity of *Saraca asoca*

Percent inhibition of the radical DPPH by *Saraca asoca* extract is mentioned in Table S1 and also in Figure S1. Sample was taken in µg/mL. To compare the results, ascorbic acid was selected as reference. The IC_{50} value of *Saraca asoca* was 115.2 µg/mL as compared to that of ascorbic acid, of which IC_{50} value was 48.21 µg/mL.

Table S1: The Ethanolic Extract of *Saraca asoca*'s (EESA) DPPH radical scavenging activity

**AA Standard**		***Saraca asoca* (Ethanolic extract)**	
**Sample (µg/mL)**	**Scavenging %**	**Sample (µg/mL)**	**Scavenging %**
0	0	0	0
20	36	20	30
40	53	40	37
60	64	60	50
80	72	80	61
100	81	100	73

AA = Ascorbic Acid, DPPH = 2, 2 – diphenylpicrylhydrazyl

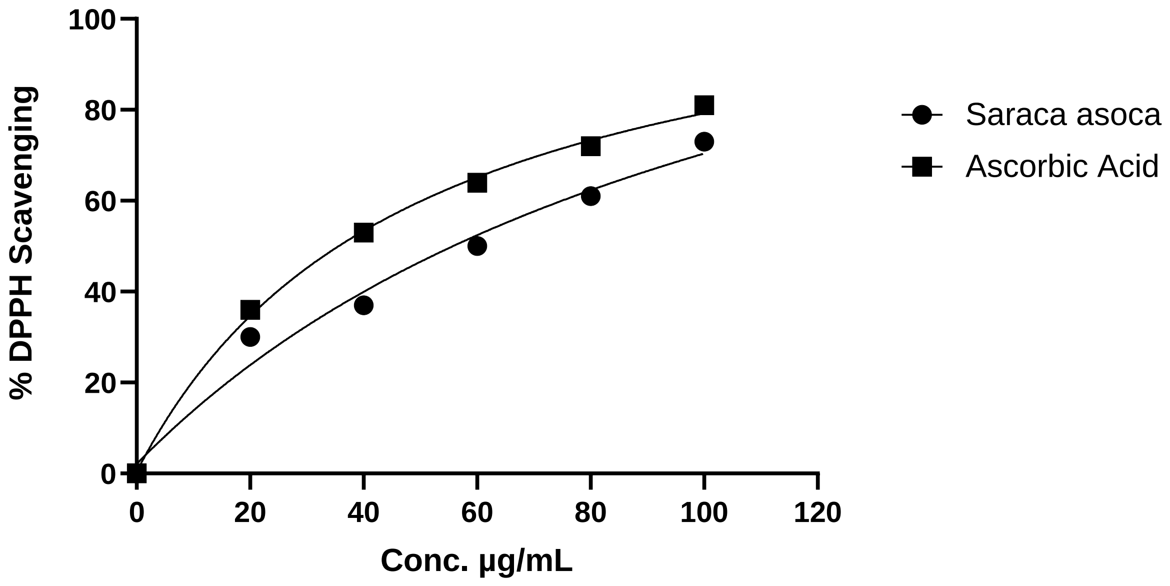

Figure S1: DPPH radical scavenging activity of *Saraca asoca* (Roxb.) Willd.
